# Engineering of Extracellular Vesicles for Targeted Delivery of Prodigiosin

**DOI:** 10.3390/biotech15010021

**Published:** 2026-03-01

**Authors:** Ivan Guryanov, Sirina Sabirova, Svetlana Batasheva, Svetlana Konnova, Arthur Khannanov, Marianna Kutyreva, Ekaterina Naumenko

**Affiliations:** 1 Institute of Fundamental Medicine and Biology, Kazan Federal University, Kremlevskaya, 18, 420008 Kazan, Russia; svbatasheva@gmail.com (S.B.); svetaka14@gmail.com (S.K.); 2 Alexander Butlerov Chemical Institute, Kazan Federal University, Kremlevskaya, 18, 420008 Kazan, Russia; arthann@gmail.com (A.K.); mkutyreva@mail.ru (M.K.)

**Keywords:** prodigiosin, extracellular vesicles, drug delivery systems, prodigiosin-induced membrane vesicles, prodigiosin encapsulating

## Abstract

The therapeutic potential of prodigiosin as a hydrophobic anticancer agent can be enhanced by various approaches, one of which is the loading of PG into extracellular vesicles. Drug distribution and stability in aqueous media play a crucial role in targeting and accumulation, thereby enabling the attainment of therapeutically effective drug concentrations. Extracellular vesicles are nano-sized, cell-derived vesicles with a lipid bilayer membrane. Extracellular vesicles can be utilized as drug carriers for both water-soluble and non-water-soluble therapeutic agents. We hypothesized that microvesicles could effectively address the current challenges of prodigiosin delivery. Several different techniques have been developed for fabricating extracellular vesicles. These include microvesicles induction by cytochalasin B treatment as well as cell cultivation in serum depleted media. In our study, prodigiosin, like cytochalasin B, demonstrated efficacy in microvesicles formation based on protein quantification and Nanoparticle Tracking Analysis. In addition, Nanoparticle Tracking Analysis showed that vesicles from mesenchymal stem cells are more stable under ultrasound exposure. Microvesicles encapsulating prodigiosin, compared to unmodified naïve ones, demonstrated slightly increased zeta potentials and hydrodynamic diameters, which probably contributed to better stability. We demonstrated that ultrasonic treatment for the loading of prodigiosin does not significantly increase the proportion of prodigiosin-positive microvesicles in comparison with microvesicles induced with prodigiosin; moreover, this method cannot be considered as optimal due to its disadvantages, such as particle aggregation. Prodigiosin-induced and prodigiosin-loaded microvesicles from mesenchymal stem cells were significantly smaller and less polydisperse in size. Overall, prodigiosin encapsulated in extracellular vesicles might be more suitable for medical and clinical applications compared to pure forms of PG due to their cell membrane compatibility.

## 1. Introduction

Cancer is a multifactorial disease, which is why its treatment requires the development of new drugs acting as multi-target inhibitors, which could be a single drug with multiple inhibitory effects or combinational therapy of more than one drug so as to simultaneously hit more than one pathway all at once [1]. From this point of view, prodigiosin is a promising candidate for the development of such therapeutics due to its multi-target action.

Prodigiosin (PG) is a red linear tripyrrole pigment that represents bioactive secondary metabolite with low molecular weight (324 Da) and molecular formula C_20_H_25_N_3_O [2,3]. This red pigment, produced by some bacteria and other microorganisms, exhibits anti-inflammatory, anti-cancer, and antioxidant activity and can be considered as a prominent therapeutic for various diseases and conditions [1].

Many studies show that PG has immunomodulatory properties [4,5]. Depending on the type of cells and conditions of the cellular environment, PG can both stimulate and suppress the immune system, selectively acting on T and B lymphocytes, tumor-associated macrophages (TAMs) and dendritic cells (TADCs), natural killer (NK) cells, and myeloid-derived suppressor cells (MDSCs) [4,5].

PG is involved in modulating and reprogramming the metabolism of various immune cells in the tumor microenvironment, which, in turn, might introduce it as an immunomodulator in cancer therapy [5]. In macrophages, prodigiosin has been shown to inhibit NADPH oxidase activation by interfering with the assembly of its cytosolic components, such as Neutrophil cytosol factor 1 (p47phox) and Ras-related C3 botulinum toxin substrate (Rac) [6]. Prodigiosin can have immunosuppressive effects, particularly on certain T cells [7]. PG can also reduce reactive oxygen species (ROS) generation induced by MC-LR (a toxin produced by cyanobacteria) by enhancing Nrf2 protein transcription factor translocation into the nucleus of HepG2 cells [8]. It has also been shown to have immunosuppressive effects and influence inflammatory processes by regulating enzymes and signaling pathways such as Cyclooxygenase-2 (COX-2) and Nuclear factor NF-kappa-B (NF-κB) [9]. PG, especially in the presence of copper ions, can damage DNA through oxidative mechanisms, leading to cell death. DNA damage occurs when the prodigiosin-copper complex intercalates DNA and promotes the formation of reactive oxygen species, which cleave both double-stranded DNA and RNA leading to inhibition of cell-cycle progression and cell death [10,11,12]. Comet assay analysis showed that PG cause a selective DNA damaged in HCT 116 and Caco-2 cancer cells [13]. PG could mediate macrophage activity and prostaglandin biosynthesis and may prevent the infiltration and M2 polarization of tumor-associated macrophages, which are often involved in promoting tumor growth [5].

Many studies have demonstrated a broad range of anticancer therapeutic benefits of PG. It selectively kills cancer cells through multiple mechanisms, including both caspase-dependent and independent induction of apoptosis, inhibiting Wnt/β-catenin signaling that accordingly causes cell-cycle arrest disrupting cancer cell metabolism. PG can interfere with cancer cell metabolism by regulating amino acid utilization and binding to proteins like glucose transporter 1 (GLUT1), which is involved in glucose uptake [14,15]. PG can raise the radio-sensitivity of cancer cells by inflammatory cascade induction followed by changes in redox tone (expressed by increase in Superoxide dismutase (SOD) and glutathione (GSH) activities and decrease in malondialdehyde (MDA) concentration), resulting in reduction in tumor growth [15]. Additionally, this drug is not excreted through ABC transporters (MDR-pumps), which are responsible for the development of chemotherapy resistance [16], so it can be used in the treatment of multiple drug-resistant cancer types.

Encapsulation has opened up opportunities for targeted drug delivery, particularly in cases where chemotherapeutic drugs have low bioavailability due to their hydrophobicity [17]. Poor water solubility of PG is a significant challenge in cancer therapy, as it can lead to poor dissolution rates and low bioavailability, requiring specialized formulation techniques to overcome these issues [1,18]. These limitations have driven extensive research into nano- and micro-encapsulation strategies, which aim to enhance its solubility, stability, and targeted delivery. It should be pointed out that research on PG encapsulation strategies is limited. The incorporation of PG into nanoparticles or nanoemulsions currently represents a key strategy to address accessibility and biocompatibility issues, thereby optimizing its therapeutic potential. Mohamed et al. proposed the zein/sodium caseinate nanoparticles for encapsulation of celecoxib and PG and demonstrated good encapsulation efficiencies (EE) and bioavailability in vitro on human breast cancer MDA-MB-231 cells [19]. Nano-encapsulation with polysaccharides like β-cyclodextrin (BCD), maltodextrin (MD), and gum arabic (GA) has been shown to significantly increase its water solubility, broadening the applicability of PG in food, pharmaceutical, and cosmetic industry [20]. Anjum et al. also proposed the encapsulation of PG into nanoparticles of soy protein isolate (SPI) and demonstrated the EE up to 89% and particle size ranged from 115.63 to 181.42 nm. Peptide-guided dendrigraft poly-l-lysines nanoparticles for targeted delivery of prodigiosin to choriocarcinoma was studied in vivo by Zhao et al. [21]. In this study, a method for targeted delivery of chemotherapeutic drugs only to cancer cells and not to normal cells in vivo using synthetic placental chondroitin sulfate-binding peptide (plCSA-BP) was investigated. Previously we demonstrated the selective cytotoxic activity of PG encapsulated in halloysite nanotubes against cancer cells, while normal cells such as human skin fibroblasts (HSF) and mesenchimal stem cells (MSCs) remained unaffected [13]. PG has also been used to graft β-cyclodextrin (CD) and chitosan (CS) magnetic nanoparticles to create an anticancer drug delivery system [22]. Fluorescence microscopy and flow cytometry confirm the specific effect of the nanocarriers on cancer cells. The results indicate that CS-MNPs exhibit higher activity and better counteract the toxic effects of prodigiosin on cancer cells than β-CD-MNPs. Mannan-coated, enzyme-sensitive, and PG-loaded magnetic nanoparticles (PG@M-MNPs) have been used for breast and liver cancer therapy and for modeling tumor-associated macrophage immunomodulation [23]. The increased selectivity of PG@M-MNPs for cancer cells with minimal impact on normal cells has also been shown. Furthermore, the immunomodulatory activity demonstrates the potential of PG@M-MNPs to alter macrophage polarization dynamics.

Another strategy for targeted delivery of PG is the formation of microcapsules from various materials, which are designed to improve the biocompatibility, stability and bioavailability of PG. Encapsulating drugs in polymeric materials creates a platform for targeted delivery of chemotherapeutic agents. Polymer delivery systems maximize the therapeutic activity and can reduce the side effects of anticancer drugs. Dozi-Nwachukwu et al. prepared PG-loaded chitosan microspheres with high encapsulation efficiency that increased with increasing drug:polymer ratio and demonstrated a decrease in the viability of a breast cancer cell line (MDA-MB-231) [24]. Polylactic-co-glycolic acid-polycaprolactone (PLGA-PCL) microspheres have also been used to load PG for targeted delivery of anticancer drugs [25].

Another promising method for encapsulating PG may be loading it into membrane vesicles. Moreover, depending on the drug’s intended application, different cell types can be used. Extracellular vesicles (EVs) is a general term used to describe a population of heterogeneous spherical particles ranging from 40 to 1000 nm in size, encapsulated by a double phospholipid layer [26] and released by all cell types [27], which transport proteins, lipids, nucleic acids, metabolites and membrane receptors of the cells from which they originate. There are three main groups of EVs: exosomes (30–160 nm), microvesicles (50–1000 nm), and apoptotic bodies (500–5000 nm).

EVs recognized as key mediators of intercellular communication and disease, are rapidly moving from basic research into clinical practice. Their capacity to transport bioactive molecules makes EVs a promising platform for diagnostics and targeted therapy, and interest in their clinical application is growing quickly [28]. EVs protect their cargo from degradation, which makes them important drug carriers for targeted drug delivery [29]. To date, no exosome- or extracellular-vesicle-based therapeutic has received final FDA approval; however, the roster of ongoing clinical trials provides hope for the introduction of such agents for the treatment of various diseases.

Cancer-cell-derived MVs tend to interact more readily with cancer-cell membranes; however, their use as drug-delivery vehicles poses safety concerns, as such MVs may promote tumor growth [30,31]. EVs from mesenchymal stem/stromal cells (MSC-EVs) can either stimulate or inhibit tumor growth in various malignancies through paracrine signaling. Some MSC-EVs have been shown to protect cancer cells from chemotherapy drugs, potentially leading to treatment failure [32], some MSC-EVs carry cargo that can suppress tumor growth by inducing apoptosis and inhibiting proliferation [33,34].

Extracellular vesicles derived from stem cells have emerged as a promising vehicle for drug delivery. EVs secreted by stem cells—including embryonic stem cells (ESCs) and MSCs—exert diverse functions, spanning embryonic development and tissue repair to cancer progression and immune modulation [28]. A recent study demonstrated that treating U266 multiple myeloma cells with MVs derived from bone marrow mesenchymal stem cells reduces their viability [35]. MSCs have been demonstrated to have immunomodulatory and regenerative functions in a variety of disease models [36]. Mesenchymal stem cells are considered an alternative treatment for rare diseases due to their immunomodulatory activity and stimulation of tissue regeneration. Their effects are driven by direct cell-mediated actions and by influences exerted through their secretome—the set of molecules and exosomes released into the medium [37]. MSC-EVs are small, cross the blood–brain barrier, and exhibit low immunogenicity with high biocompatibility [38]. In preclinical models, MSC-derived exosomes enhanced wound healing, promoted bone regeneration, and facilitated cartilage repair by modulating inflammation, angiogenesis, cell proliferation, and matrix synthesis [39]. Beyond MSCs, common sources of EVs include tumor cells, human embryonic kidney 293 (HEK 293) cells, and dendritic cells (DCs). HEK 293 cells are favored due to their high transfection efficiency, rapid growth, and ease of culture. HEK 293 cells produce large quantities of exosomes, making them an ideal model for obtaining drug-loaded exosomes.

There are no reports yet on attempts to load PG into membrane vesicles, so in our study for the first time, we produced MVs as a prototype of an EV-based drug delivery system, loaded MVs with PG, and performed a comparative analysis of the size, charge, and PG content in MVs induced by cytochalasin B and PG.

## 2. Materials and Methods

### 2.1. Cells and Culture Conditions

MSCs were isolated from rat adipose tissue by digestion using 0.2% collagenase from crab hepatopancreas (Biolot, St. Petersburg, Russia), according to the previously developed protocol [40]. The HEK 293 cell line isolated from the kidney of a human embryo (ATCC # CRL-1573), was obtained from the American Type Culture Collection (ATCC, Manassas, VA, USA). Cells were cultured using two different types of cell culture media, α-MEM (Alpha-Minimum Essential Medium) and DMEM/F12 (Dulbecco’s Modified Eagle Medium/Nutrient Mixture F-12) (PanEco, Moscow, Russia) with 10% FBS (HyClone, Logan, UT, USA), 2 mM of L-glutamine, penicillin (100 U/mL) and streptomycin (100 μg/mL) antibiotics (PanEco, Moscow, Russia). Cells were incubated at 37 °C and 5% CO_2_ and maintained according to the standard protocols. Cell morphology was examined by an Axio Observer.Z1 (CarlZeiss, Jena, Germany) microscope and Axio Vision Rel. 4.8 software.

### 2.2. Isolation of Prodigiosin and Cytochalasin B-Induced Membrane Vesicles

PG was isolated by acidic ethanol extraction from S. marcescens bacterial biomass and purified as described previously [3]. PG was dissolved in DMSO for all experiments. PG identity and indicative purity were assessed by UV–Vis spectroscopy. Vesicles were obtained from MSCs (MSC CBiMVs) and HEK 293 cells (HEK CBiMVs) using cytochalasin B (cytochalasin B from *Drechslera dematioidea*, #C6762-5MG, Sigma-Aldrich, St. Louis, MO, USA) as described previously [41] or PG. When cell culture reached 85–90% confluency, the medium was removed, the culture was washed twice with phosphate-buffered saline (PBS) and the cells were detached with 0.25% trypsin-EDTA solution (PanEco, Moscow, Russia). Then the cells were washed with PBS, centrifuged, resuspended and incubated in modified serum-free Dulbecco’s Modified Eagle Medium (DMEM) (4 × 10^6^ cells in 2 mL) containing 10 μg/mL cytochalasin B (Sigma-Aldrich, St. Louis, MO, USA) or PG (50 ng/mL and 250 ng/mL, 100 and 500 ng PG, respectively, per experiment) for 30 min at 37 °C in a humidified atmosphere with 5% CO_2_. During incubation cells were vortexed twice: after 15 min of incubation (for 30 s) and for 60 s after 30 min of incubation. Next, a series of subsequent centrifugations were carried out: RCF 317× *g* for 10 min (supernatant was collected), RCF 317× *g* for 10 min (supernatant was collected), and RCF 13,500× *g* for 15 min (supernatant was discarded).

### 2.3. Loading of MVs with Prodigiosin

The samples obtained in the previous step were divided into two types of probes—for additional loading with prodigiosin/sonification and without it. To load MV with PG a mixture of MVs with PG (250 ng) was sonicated using an ultrasonic homogenizer Bandelin SONOPULS HD 2200, 70 W with booster horn SH 213 G, tip TT13 (BANDELIN electronic GmbH & Co. KG, Berlin, Germany) with an amplitude of 45% for six cycles, each cycle included six on/off periods of 30 s with a cooling period of 2 min between cycles [42]. After ultrasonication, the samples were filtered through 5 μm membrane filters and precipitated at RCF 13,500× *g* for 15 min. After resuspending the precipitate in PBS, the MVs suspension was used for further analysis.

### 2.4. Protein Concentration Measurement

The CBiMVs and PGiMVs were incubated in lysing solution (50 mM Tris-HCl pH 7.4, 1% NP 40, 0.5% sodium deoxychalate, 0.1% SDS, 150 mM NaCl, 2 mM EDTA, and 1 mM PMSF) for 30 min on ice. Then the resulting mixture was centrifuged at RCF 13,500× *g* for 15 min at 4 °C. Total protein concentration was determined in supernatant using the Pierce™ BCA Protein Assay Kit (ThermoScientific, Waltham, MA, USA) according to the manufacturer’s instructions.

### 2.5. Hydrodynamic Diameter (Dh) and Zeta Potential (ζ) Analysis

Dh and zeta potential of EVs were measured in water at 25 °C by dynamic light scattering and laser Doppler velocimetry using Zetasizer Nano ZS instrument (Malvern, UK) and standard plastic U-shaped cells.

### 2.6. Microscopy

For the scanning electron microscopy (SEM) analysis, suspensions of microvesicles (MVs) were dehydrated through a series of ethanol solutions ranging from 10% to 96%. The suspensions were then placed onto clean glass coverslips in a 24-well plate and subjected to centrifugation at 3000× *g* for 30 min. Following this, the MVs were fixed with 10% formalin for 15 min, washed twice with ultrapure water, dehydrated using an ethanol gradient from 30% to 100%, and air-dried for 24 h. Visualization was carried out using an Auriga field-emission scanning electron microscope (Carl Zeiss) with an Inlens secondary electron detector at an accelerating voltage of 5 keV and an electron beam current of 300 pA. Prior to imaging, samples were sputter-coated with gold to facilitate electron conduction, with a coating thickness of approximately 10 nm.

Dark-field images and reflectance spectra were obtained using an Olympus BX51 upright microscope (Olympus, Tokyo, Japan). The setup included a CytoViva^®^ darkfield condenser, a Fiber-Lite DC-950 halogen light source (150 W) (Dolan Jenner Industries Inc. Boxborough, MA, USA), and a ProScan III control unit (Prior Scientific, Inc., Rockland, MA, USA). Hyperspectral imaging within the wavelength range of 400 to 1000 nm, with a fixed exposure time of 0.25 s, was performed using a Specim V10E spectrograph (Spectral Imaging Ltd., Oulu, Finland) coupled with a CCD camera. The hyperspectral data were corrected for illumination variations and analyzed with ENVI software v. 4.8 (Harris Geospatial Solutions, Broomfield, CO, USA). This technique is highly effective for label-free visualization of micro- and nano-sized inorganic and biopolymer particles [43,44].

### 2.7. Flow Cytometry

MVs were analyzed using flow cytometry. A mixture of calibration particles of 0.22, 0.45, 0.88, 1.34, and 3.4 μm (Cat. No. PPS-6K, Spherotech, Lake Forest, IL, USA) was used for the calibration of the CytoFLEX S (Beckman Coulter, Brea, CA, USA). Analysis of the yield of MVs and PG capacity was performed using a CytoFLEX S (Beckman Coulter, USA) flow cytometer. Each sample was recorded within 60 s. Prodigiosin with inherent red color (absorption at ~535 nm) and fluorescent properties (excitation peak at 543 nm, emission peak at 570 nm), ref. [45] was detected compared to Phycoerythrin (PE) standard (excitation peak at 565 nm and an emission peak at 578).

### 2.8. Nanoparticle Tracking Analysis (NTA)

Colloidal properties were analyzed using Nanoparticle Tracking Analysis (NTA) with a NanoSight LM-10 instrument (Malvern Panalytical, Malvern, UK). The system employed a C11440-50B CMOS camera paired with an FL-280 Hamamatsu Photonics image capture sensor (Hamamatsu Photonics, Shizuoka, Japan) as the detector. Measurements were conducted in a specialized aqueous solution cuvette, which was equipped with a 405 nm laser (version CD, S/N 2990491) and sealed with a Kalrez ring to prevent evaporation. The temperature during measurements was monitored using an OMEGA HH804 contact thermometer (Engineering, Inc., Stamford, CT, USA). Samples were injected into the measurement chamber using a 1 mL glass, two-piece syringe (tuberculin) via a Luer connector (Hamilton Company, Reno, NV, USA). To enhance statistical reliability, samples were circulated through the chamber utilizing a piezoelectric dispenser. Each sample was measured in six consecutive runs, with recordings taken sequentially for 60 s each. The recorded videos were analyzed using NTA 2.3 software (build 0033), and data processing was performed with the OriginPro 8.7 software package; a Gaussian fit was applied throughout, following the approach described previously [46,47,48,49].

### 2.9. Statistical Analysis

Statistical analysis was performed using Student’s *t*-test (GraphPad Prism 8.0.1 Software, San Diego, CA, USA) at a significance level of 0.05.

## 3. Results

### 3.1. Quantitative Characterization of Microvesicles

Table 1 represents all samples and their fabrication conditions (Table 1).

**Figure 1:**
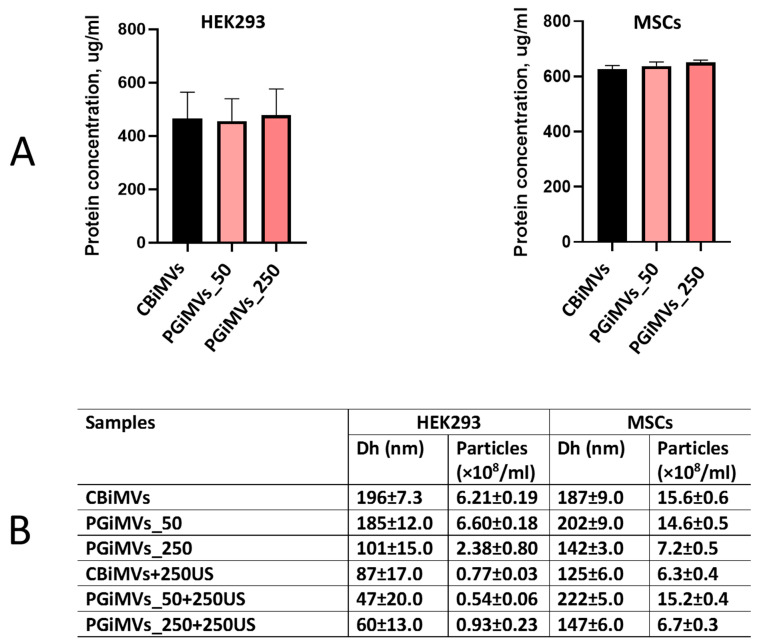
Protein concentration measurement (**A**) and determination of the size and concentration of MVs in NTA (**B**): recording time was sequential and amounted to 60 s, laser—405 nm; camera level—10; detection threshold—7; slider shutter—1206; camera Shutter (ms)—8.75 ms, slider gain—245; syringe pump speed—50; each sample was detected sequentially six times.

Quantitative BCA Protein Assay and Nanoparticle tracking analysis (NTA) was used to quantify colloidal properties including the number of MVs in a sample volume and their size distribution. Quantitative analysis of MVs protein content showed that there were no significant differences between cytochalasin B and PG in the number of MVs induced from HEK 293 and MSCs (Figure 1A).

As can be seen from the particle size distribution in the table (Figure 1B, samples CBiMVs + 250US, PGiMVs_50 + 250US and PGiMVs_250 + 250US), ultrasound can provoke the disruption of membrane integrity of the cytochalasin B and PG induced MVs (CBiMVs and PGiMVs). Then they self-assemble into more stable, smaller aggregates in case of HEK 293 cells. Moreover, a significant decrease in the overall particle concentration indicates that some vesicle components remain as individual molecules in solution. Characteristically, vesicles derived from MSCs are more resistant to external physical stimulation, which is generally preferable for targeted delivery systems. All graphs demonstrating the size and concentration of MVs determined in NTA are presented in Supplementary Materials (Figures S2 and S3).

### 3.2. Analysis of MVs Hydrodynamic Diameter and Zeta Potential

**Figure 2:**
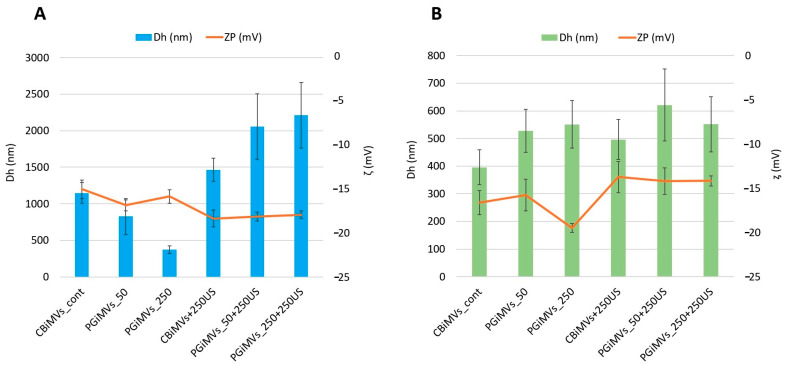
Hydrodynamic diameters (Dh, nm) and zeta potentials (ζ, mV) of MVs obtained by induction with cytochalasin B (CBiMVs) and prodigiosin (PGiMVs) from HEK 293 (**A**) and rat MSC (**B**).

The typical characteristics of EVs including exosomes and MVs are their size and shape, as these parameters can affect other properties such as colloidal stability [50]. To characterize particle Dh and zeta potential of different types of MVs developed in our study, the hydrodynamic Dh and zeta potentials of MVs were determined by dynamic light scattering (DLS) and laser Doppler velocimetry (Figure 2). We suggested that PG as a cargo and inducer of MVs production as well as method of loading can influence the surface charge distribution and change the Dh, surface charge, and stability of the synthesized MVs. Overall, Zeta potential acts as a crucial indicator of colloidal stability.

Values of the zeta potential of MVs induced from MSCs and HEK 293 cells range from −13.75 mV to −19.5 mV (Figure 2). These values significantly increase after ultrasound treatment with the addition of PG for HEK 293 microvesicles, while they decrease slightly for MVs from MSCs. zeta potential for HEK 293-derived Cytochalasin B induced MVs (CBiMVs) was −15.02 mV with a hydrodynamic diameter of 1147 nm. After addition of 250 ng PG and US treatment, the hydrodynamic diameter of the MV increased by 27%, reaching a value of 1463 nm, the zeta potential of CBiMV+250 was −18.35 mV. This value of Zeta potential indicates good colloidal stability of microvesicles.

**Figure 3:**
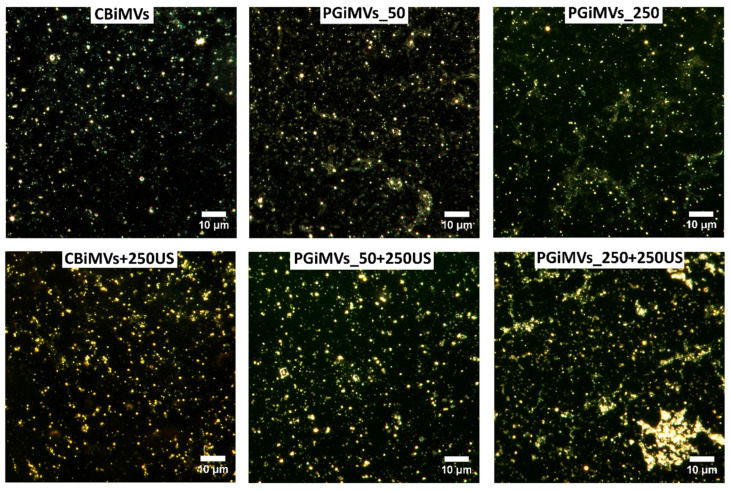
CytoViva darkfield hyperspectral images (100×) of MVs induced by Cytochalasin-B or prodigiosin from HEK 293 cells.

The hydrodynamic diameter of PG-induced vesicles from HEK 293 cells varied significantly. At 50 ng of PG, the vesicle hydrodynamic diameters were similar to those of CBiMVs, reaching a value of 826 nm and a zeta potential of −16.83 mV. The smallest microvesicles from HEK 293 cells were induced using 250 ng of PG (Dh—373 nm, ζ −15.83 mV). Loading of PG and US of PG-induced microvesicles resulted in their increase by more than 2 folds in PGiMVs_50 + 250, Dh—2056 nm (ζ −18.11 mV) and PGiMVs_250 + 250, Dh—2211 nm (ζ −17.91 mV). In comparison to HEK 293-derived CBiMVs, CBiMVs from MSCs had significantly lower Dh (about 396 nm) and slightly higher zeta potential values of −16.63 mV. Fivefold increase in the amount of PG for MV induction leads to a slight increase in the size of 551 nm PGiMVs_250 versus 527 nm PGiMVs_50 and an increase in the zeta potential from −15.76 mV to −19.5 mV. Figure 2 shows that after ultrasonic loading of the drug into PG-induced MVs from MSCs the zeta potential decreases from −15.76 mV (PGiMVs_50) to −14.2 mV (PGiMVs_50 + 250US) and from −19.76 mV (PGiMVs_250) to −14.16 mV (PGiMVs_250 + 250US). Moreover, the Dh changed only in PGiMVs_50 to 621 nm. The increase in the polydispersity index is possibly the result of an increase in the number of aggregates from a multitude of MVs. This view is supported by CytoViva darkfield hyperspectral images (Figure 3). Aggregation became more intensive upon the addition of PG to the MVs suspension, as well as the partial release of PG from the MVs during ultrasonic treatment. The highest polydispersity index (close to 1), is demonstrated by samples PGiMVs_50 + 250US and PGiMVs_250 + 250US.

### 3.3. Visualization of MVs

Dark-field photographs of different samples of MVs demonstrate the abundance and shape of MVs (Figure 3). Dark-field microscopy with hyperspectral analysis demonstrated different reflectance spectra for control and PG-loaded microvesicles (Figure S4). The reflectance spectrum of control vesicles was lower in intensity than that of PG-loaded microvesicles and showed a gradual decrease from 440 to 460 nm to 1000 nm. The reflectance spectrum of PG-loaded MVs demonstrated a prominent peak around 565 nm, the position of which was very close to that of the known PG fluorescence emission peak at about 560 nm [51,52]. Thus, hyperspectral analysis additionally confirmed the presence of PG in PG-loaded microvesicles.

**Figure 4:**
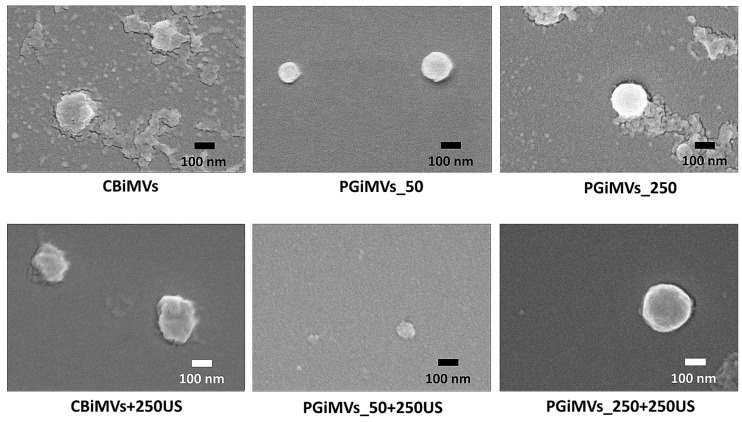
Scanning electron microscopy (SEM) images of Cytochalasin B and Prodigiosin induced microvesicles from MSC.

The SEM image illustrates the surface topography of the microvesicles, which is generated by scanning the sample with a focused electron beam and detecting the secondary electrons emitted by the atoms within the targeted area. SEM enabled the visualization of the morphology of individual microvesicles produced after induction with PG and cytochalasin B, both with and without ultrasound treatment (Figure 4). It should be noted that agglomeration was observed as a result of the drying process prior to SEM analysis.

### 3.4. Flow Cytometry Analysis

**Figure 5:**
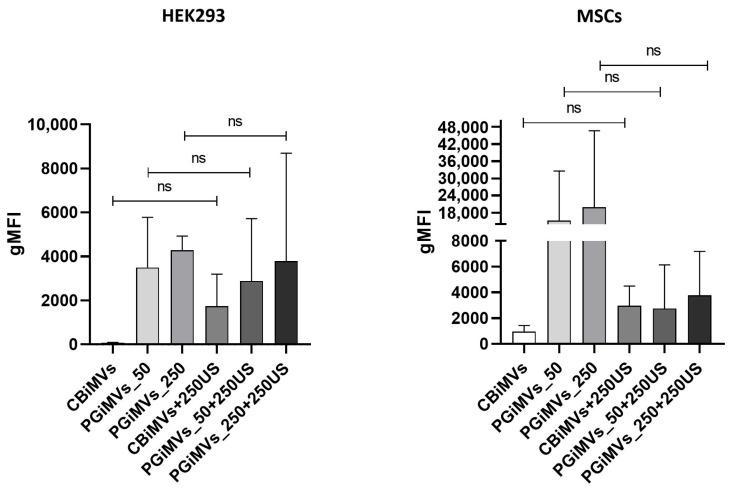
Flow cytometry (FCM) analysis of a Cytochalasin B and prodigiosin induced microvesicles from HEK 293 and MSCs. All values are means ± SD; “ns” indicates non-significant.

Different populations of MVs are distinguished in flow cytometry by their varied sizes, morphologies, and PG contents (Figure 5). Flow cytometry analysis showed that the gMFI of PG in HEK 293-derived microvesicles did not differ significantly between vesicles before and after additional loading with 250 ng/mL PG for any induction method (paired two-tailed *t*-test, *n* = 3 independent experiments: CBiMVs vs. CBiMVs + 250US, *p* = 0.40; PGiMVs_50 vs. PGiMVs_50 + 250US, *p* = 0.80; PGiMVs_250 vs. PGiMVs_250 + 250US, *p* = 0.86). For MSCs-derived microvesicles, only two independent experiments were performed (*n* = 2). Additional loading with 250 μM PG tended to increase fluorescence of CBiMVs approximately three-fold and to decrease fluorescence of PGiMVs_50 approximately six–seven-fold in both experiments, whereas the effect on PGiMVs_250 was inconsistent between experiments. Due to the very small sample size (*n* = 2), these observations are descriptive and did not reach statistical significance in paired two-tailed *t*-tests.

Overlaid histograms show prodigiosin-PE signal in CBiMVs and CBiMVs + 250US, PGiMVs_50 and PGiMVs_50 + 250US, and PGiMVs_250 and PGiMVs_250 + 250US. Each row (R1–R2) represents an independent experiment. The vertical marker indicates the gate used to define prodigiosin-positive (PG^+^) microvesicles; the corresponding percentage of PG^+^ events is shown on each plot. In both cell types, the proportion of PG^+^ vesicles remains very low under most conditions and varies significantly between replicates. For PGiMVs_50 and PGiMVs_250, the additional load of PG does not significantly increase the proportion of PG^+^ microvesicles: the histograms for the two conditions almost completely overlap (Figure S5). Collectively with quantitative analysis (gMFI), these data indicate that under the induction and loading conditions used, the efficiency of PG accumulation in vesicles remains low and highly variable. The different amount of PG in microvesicles obtained from different cells is primarily due to different cellular uptake of PG.

## 4. Discussion

Packaging therapeutic agents into membrane vesicles offers several advantages over other delivery systems, but the most important one is the increased bioavailability of the administered compounds due to the affinity of microvesicles for cell membranes. Membrane vesicles can fuse with the cell membrane, combining their lipid layers and releasing their contents into the cell [53]. The vesicles are equipped with specific proteins (in particular, SNARE proteins) that recognize and bind to corresponding proteins on the cell membrane. Upon contact, the lipid layers mix and pores form, allowing the contents to enter the cell. SNAREs form an extremely stable complex, allowing membrane tethering and subsequent MVs fusion with the plasma membrane [54]. An advantage of microvesicles as a delivery system is the high efficiency of delivery of lipophilic compounds [55].

Despite the advantages of high drug-loading efficiency and continuous drug release, sonication may have adverse effects on the structure of exosomes, causing changes in the spherical shape and hydrophobic drug-loading efficiency, as well as inducing exosome aggregation. Nevertheless, sonication remains a popular method for developing novel exosome-driven drug-delivery systems due to its great potential for optimizing drug delivery [56].

In many cases, the sonication technique yields a higher loading efficiency compared to co-incubation and electroporation. For example, a study involving the loading of paclitaxel into macrophage-derived exosomes reported a maximum loading capacity of 28.3% with sonication, whereas incubation and electroporation resulted in only 1.4% and 5.3%, respectively [57]. Similarly, when examining gemcitabine loading, sonicated exosomes exhibited greater efficiency (11.7 ± 3.7%) than those loaded by incubation alone (2.8 ± 0.7%) [58]. In our work, flow cytometry analysis revealed that loading PG did not significantly increase the proportion of PG-positive MVs compared to MVs induced with PG alone. Additionally, the drawbacks of the ultrasound method, such as particle aggregation, prevent us from considering this approach optimal for PG loading.

Extracellular vesicles (EVs) are characterized by their physical attributes—including particle size, concentration, surface charge, and density—as well as their biological properties, such as the composition of internal and external biomolecules, including membrane-associated antigens [59]. Techniques such as electron microscopy [60], atomic force microscopy [61], dynamic light scattering (DLS), flow cytometry [62], and NTA [63] can be used to measure the size, shape, and density of EV particles. To analyze the size and amount of MVs obtained in our study we used NTA which has become the gold standard for characterizing the physical properties of MVs and other extracellular vesicles. Generally, NTA is well suited to study exosomes [64]. This method provides a unique opportunity to rapidly analyze the size, concentration, and distribution of nanoparticles in suspension, which is critical for their study as biomarkers and functional agents. NTA is the first and essential method for analyzing MVs after isolation. It allows determination of the particle-size profile (the peak for MVs is typically in the 100–500 nm range) and precise particle concentration. This is essential for standardizing experiments and comparing the efficiency of different isolation protocols (e.g., ultracentrifugation vs. chromatography) [65,66]. When studying the mechanisms of MV formation or their effects on target cells, it is necessary to accurately dose the amount of vesicles added to the experiment. NTA allows for the addition of a precise number of particles to the cell culture rather than an arbitrary volume of the sample, significantly improving the reproducibility of the results. So NTA in this study allowed to evaluate particle size distribution and stability depending on the conditions of MVs formation. These data were also supported by the protein concentration data, and taken together, NTA and protein concentration analysis allowed us to determine the concentration of MVs in solution.

Examining the electrical properties of small EVs, particularly their surface charge distribution, could contribute to the development of essential new technologies in the rapidly expanding field of EVs research [67,68]. zeta potential is a widely used approach for assessing the surface potential of EVs, serving as an indicator of surface charge and colloidal stability, which are affected by surface chemistry, bioconjugation, and the specific theoretical model employed [50]. The surface of microvesicles has a negative charge, which is due to the presence of phospholipids with negatively charged groups on the outer side of the membrane, such as phosphatidylserine [50,69]. Prodigiosin possesses a specific charge and hydrophobicity that can influence the surface charge of vesicles, altering their zeta potential. It contains three pyrrole rings and one propionic group, but its charge is not fixed but rather pH-dependent. The polydispersity index (PDI) is a metric measured by a Zetasizer instrument and used to characterize Dh distribution in a sample. A low PDI value indicates a narrow, uniform size distribution, while a high PDI value indicates a broad, non-uniform distribution. Zetasizer instruments, manufactured by companies such as Malvern Panalytical, use techniques such as dynamic light scattering to measure PDI, which is critical for applications in pharmaceuticals, paints, inks, and other industries [70]. Nanoparticles with a zeta potential between −10 and +10 mV are considered approximately neutral [71], while nanoparticles with zeta potentials of greater than +30 mV or less than −30 mV are considered strongly cationic and strongly anionic, respectively. In our study MVs from MSCs and HEK 293 cells exhibited zeta potentials between −13.75 mV and −19.5 mV. After ultrasound treatment with PG, the zeta potential significantly increases for HEK 293-derived vesicles, while it slightly decreases for MSC-derived MVs. Specifically, Cytochalasin B-induced MVs from HEK 293 (CBiMVs) had a zeta potential of −15.02 mV with a hydrodynamic diameter of 1147 nm. Following the addition of 250 ng PG and ultrasound, their hydrodynamic diameter increased by 27% to 1463 nm (CBiMVs + 250US), and the zeta potential shifted to −18.35 mV, indicating maintained colloidal stability. Specifically, Cytochalasin B-induced MVs from HEK 293 (CBiMVs) had a zeta potential of −15.02 mV with a hydrodynamic diameter of 1147 nm. Following the addition of 250 ng PG and ultrasound, their hydrodynamic diameter increased by 27% to 1463 nm, and the zeta potential shifted to −18.35 mV, indicating maintained colloidal stability. Considering methodologies, it is important to recognize that Dynamic Light Scattering (DLS) relies on correlation functions and provides an average hydrodynamic diameter across the entire particle population. As a result, these measurements do not reflect the true size of individual microvesicles. Conversely, Nanoparticle Tracking Analysis (NTA) measures size based on the discrete tracking of individual particles, offering a more accurate determination of particle dimensions. Based on these measurements, we propose that the microvesicles generated in this study possess the physicochemical characteristics necessary to potentially penetrate tumor tissue through the Enhanced Permeability and Retention (EPR) effect.

## 5. Conclusions

The demonstrated anticancer activity of prodigiosin (PG), supported by multiple independent studies, and its immunomodulatory properties, positions PG as a versatile multimodal anticancer agent. Microvesicles (MVs) could effectively address the current challenges of prodigiosin delivery because phosphatidylserine on the inner side of the MV membrane serves as an “eat-me” signal recognized by immune cells, facilitating targeted uptake. The variation in PG content within microvesicles derived from different cell types is primarily due to differences in cellular PG uptake. Additionally, the size, quantity, and PG concentration of MVs are mainly influenced by cell type and experimental conditions.

Quantitative protein analysis showed no significant differences in the number of MVs induced from HEK293 and MSCs with cytochalasin B and PG stimulation. Overall, PG-induced and PG-loaded MVs from MSCs were significantly smaller and less polydisperse in size compared to those from other sources. The highest polydispersity index (close to 1) was observed in samples PGiMVs_50 + 250US and PGiMVs_250 + 250US.

Furthermore, cytochalasin induced MVs from HEK293 cells loaded with prodigiosin (CBiMVs + 250US) and MVs induced from MSCs with 250 ng PG (PGiMVs_250) demonstrate excellent electrostatic stability, owing to strong repulsive forces between charged particles. Flow cytometry analysis revealed that ultrasound (US) stimulation during MV production does not significantly increase PG loading or the proportion of PG-positive microvesicles. The selected method of inducing MVs via cell incubation with PG allows for the production of moderately stable vesicles with defined zeta potentials and sizes. Notably, MSC-derived MVs tend to be more resistant to external physical stimuli, which is often favorable for targeted delivery. Post-ultrasound treatment resulted in minor size variations, especially in MSC-derived MVs, which maintained better stability compared to those derived from HEK293 cells.

Currently, the analytical techniques and methods employed do not provide sufficient resolution to definitively determine the precise localization of prodigiosin within the microvesicles. Consequently, at this stage, PG-loaded microvesicles should be regarded as microvesicles associated with prodigiosin. The question of the exact intravesicular localization of prodigiosin within the microvesicle structure will be addressed and clarified in subsequent investigations. Future research will also be focused on optimizing MV loading parameters, such as PG concentration, incubation time, and potential additional methods like water bath heating or extended post-ultrasound incubation. These efforts aim to enhance loading efficiency and thoroughly evaluate the anticancer efficacy of PG-loaded microvesicles.

## Figures and Tables

**Table 1:** Samples of microvesicles and conditions of their fabrication.

Abbreviation of Samples	Conditions of Fabrication
CBiMVs	Cytochalasin B-induced microvesicles
CBiMVs + 250US	Cytochalasin B-induced microvesicles sonicated in the presence of 250 ng/mL prodigiosin
PGiMVs_50	Microvesicles obtained by treating cells with 50 ng/mL prodigiosin
PGiMVs_50 + 250US	Microvesicles obtained by treating cells with 50 ng/mL prodigiosin sonicated in the presence of 250 ng/mL prodigiosin
PGiMVs_250	Microvesicles obtained by treating cells with 250 ng/mL prodigiosin
PGiMVs_250 + 250US	Microvesicles obtained by treating cells with 250 ng/mL prodigiosin sonicated in the presence of 250 ng/mL prodigiosin

## Data Availability

The original contributions presented in this study are included in the article/Supplementary Material. Further inquiries can be directed to the corresponding author(s).

## Supplementary Materials

The following supporting information can be downloaded at: https://www.mdpi.com/article/10.3390/biotech15010021/s1, Figure S1: Optical spectrum of prodigiosin; Figure S2: Determination of the size and concentration of MVs isolated from HEK293 cells using Nanoparticle tracking analysis (NTA): recording time was sequential and amounted to 60 s, laser—405 nm; camera level—10; detection threshold—7; slider shutter—1206; camera Shutter (ms)—8.75 ms, slider gain—245; syringe pump speed—50; each sample was detected sequentially six times. Each sample was detected sequentially six times; the recording time was sequential and amounted to 60 s; Figure S3: Determination of the size and concentration of MVs isolated from MSCs using Nanoparticle tracking analysis (NTA): recording time was sequential and amounted to 60 s, laser—405 nm; camera level—10; detection threshold—7; slider shutter—1206; camera Shutter (ms)—8.75 ms, slider gain—245; syringe pump speed—50; each sample was detected sequentially six times; the recording time was sequential and amounted to 60 s; Figure S4: The hyperspectral analysis confirmed the presence of PG in PG-loaded microvesicles. The reflectance spectrum of PG-loaded microvesicles demonstrated a prominent peak around 565 nm, the position of which was very close to that of the known PG fluorescence emission peak at about 560 nm; Figure S5: Representative histograms assessed by flow cytometry analysis of prodigiosin content in induced microvesicles obtained from HEK293. The results of three replicates are presented. The percentages reflect the number of induced microvesicles loaded with prodiogisin; Figure S6: Representative histograms assessed by flow cytometry analysis of prodigiosin content in induced microvesicles obtained from MSCs. The results of two replicates are presented. The percentages reflect the number of induced microvesicles loaded with prodiogisin.
