# Supplementary material for: Engineering of Extracellular Vesicles for Targeted Delivery of Prodigiosin
Source: BioTech (Basel). 2026 Mar 1;15(1):21. doi: 10.3390/biotech15010021 (PMC13024301; doi:10.3390/biotech15010021)
Supplement: Supplementary file 1 [file biotech-15-00021-s001.zip › biotech-4106601-supplementary.pdf]

Supplementary Materials: Engineering of extracellular vesicles for targeted delivery of prodigiosin

Ivan Guryanov, Sirina Sabirova, Svetlana Batasheva, Svetlana Konnova, Arthur Khannanov, Marianna Kutyreva, and Ekaterina Naumenko

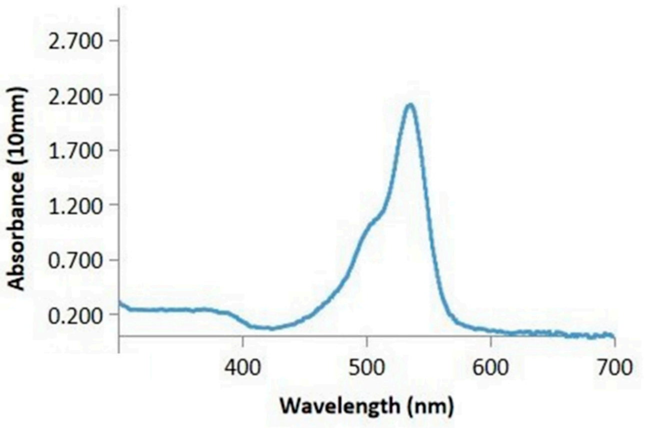

Figure S1. Optical spectrum of prodigiosin.

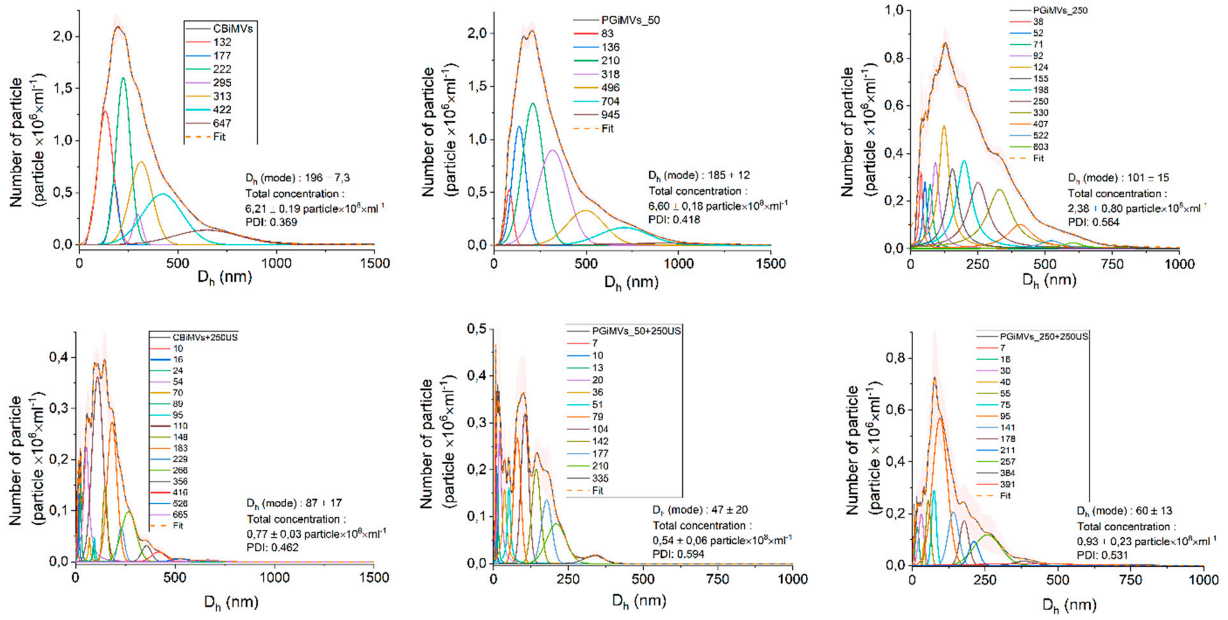

Figure S2. Determination of the size and concentration of MVs isolated from HEK293 cells using Nanoparticle tracking analysis (NTA): recording time was sequential and amounted to 60 s, laser—405 nm; camera level—10; detection threshold—7; slider shutter—1206; camera Shutter (ms) - 8.75 ms, slider gain—245; syringe pump speed—50; each sample was detected sequentially six times. Each sample was detected sequentially six times; the recording time was sequential and amounted to 60 s.

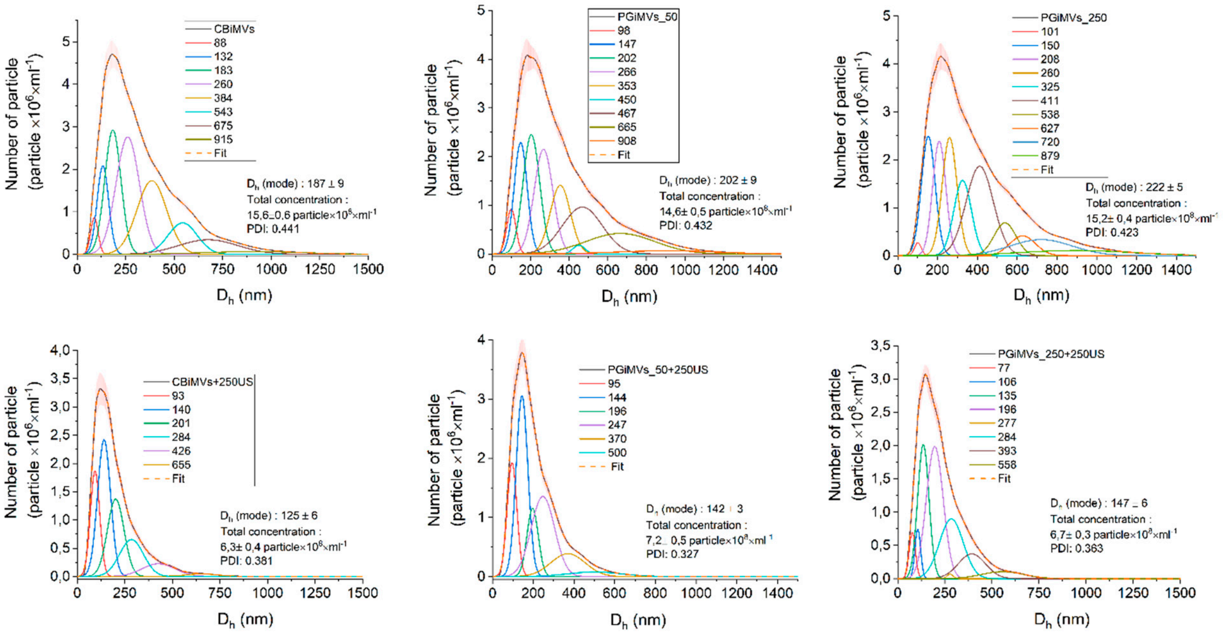

Figure S3. Determination of the size and concentration of MVs isolated from MSCs using Nanoparticle tracking analysis (NTA): recording time was sequential and amounted to 60 s, laser—405 nm; camera level—10; detection threshold—7; slider shutter—1206; camera Shutter (ms) - 8.75 ms, slider gain—245; syringe pump speed—50; each sample was detected sequentially six times; the recording time was sequential and amounted to 60 s.

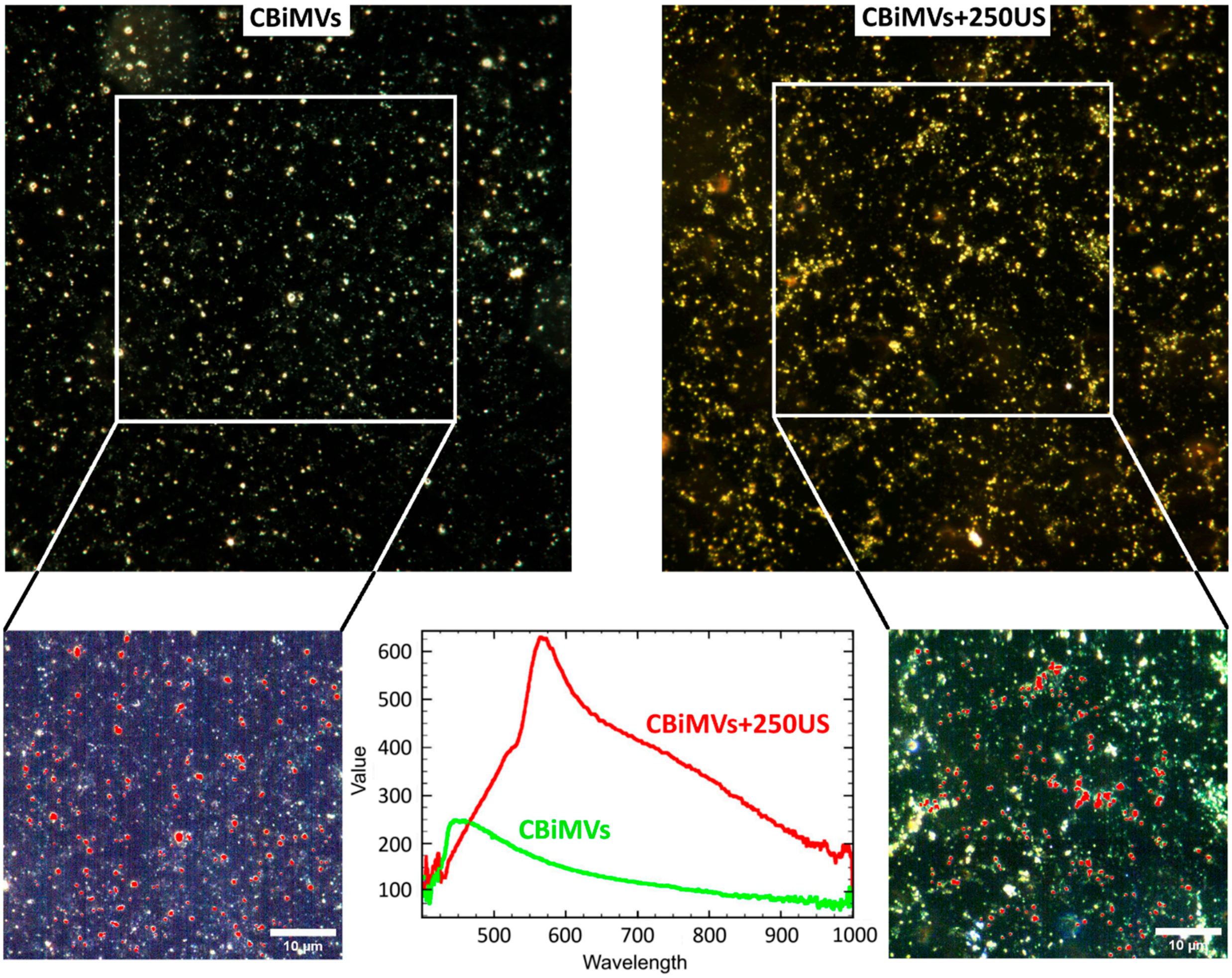

Figure S4. The hyperspectral analysis confirmed the presence of PG in PG-loaded microvesicles. The reflectance spectrum of PG-loaded microvesicles demonstrated a prominent peak around 565 nm, the position of which was very close to that of the known PG fluorescence emission peak at about 560 nm.

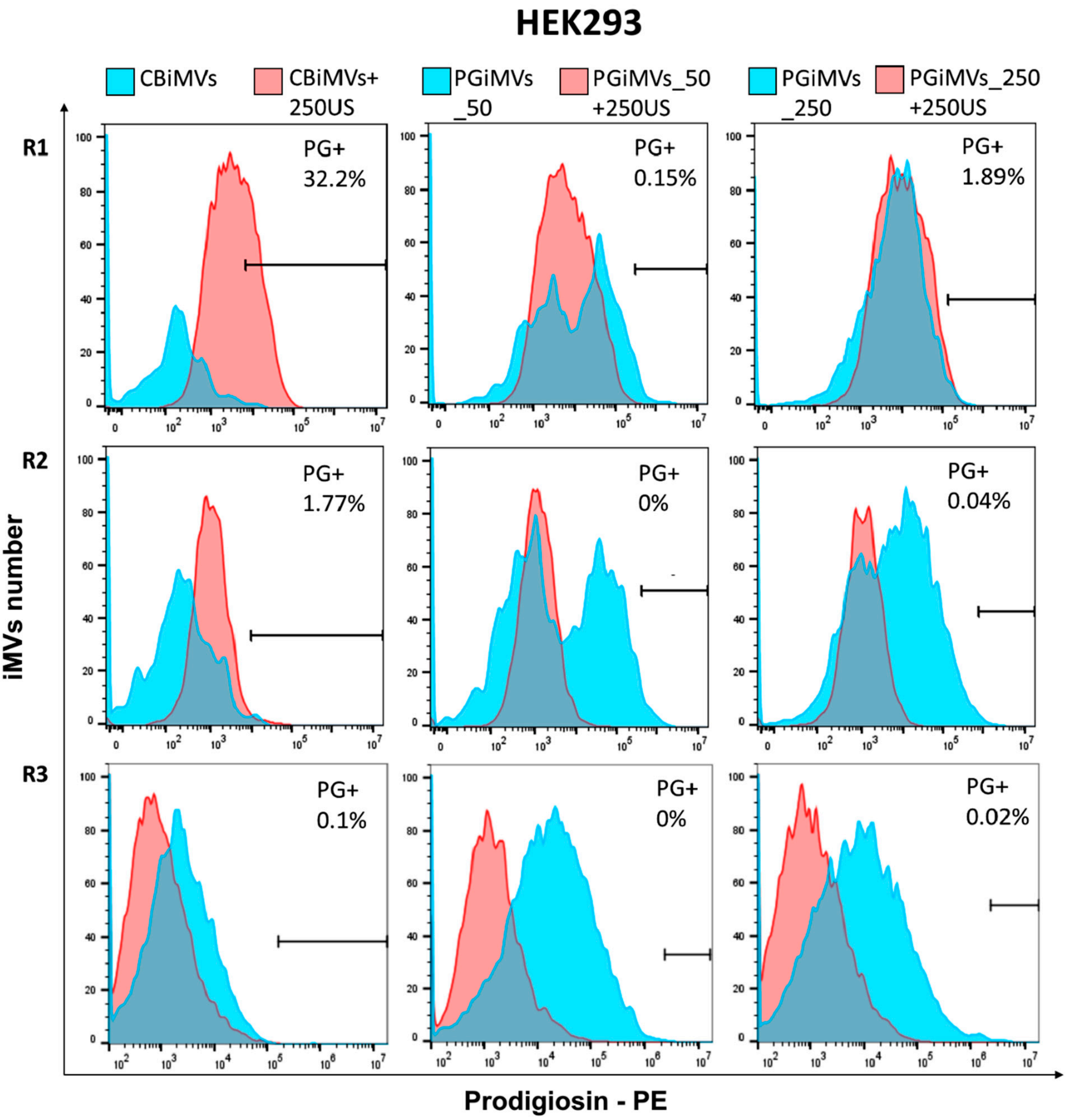

Figure S5. Representative histograms assessed by flow cytometry analysis of prodigiosin content in induced microvesicles obtained from HEK293. The results of three replicates are presented. The percentages reflect the number of induced microvesicles loaded with prodiogisin.

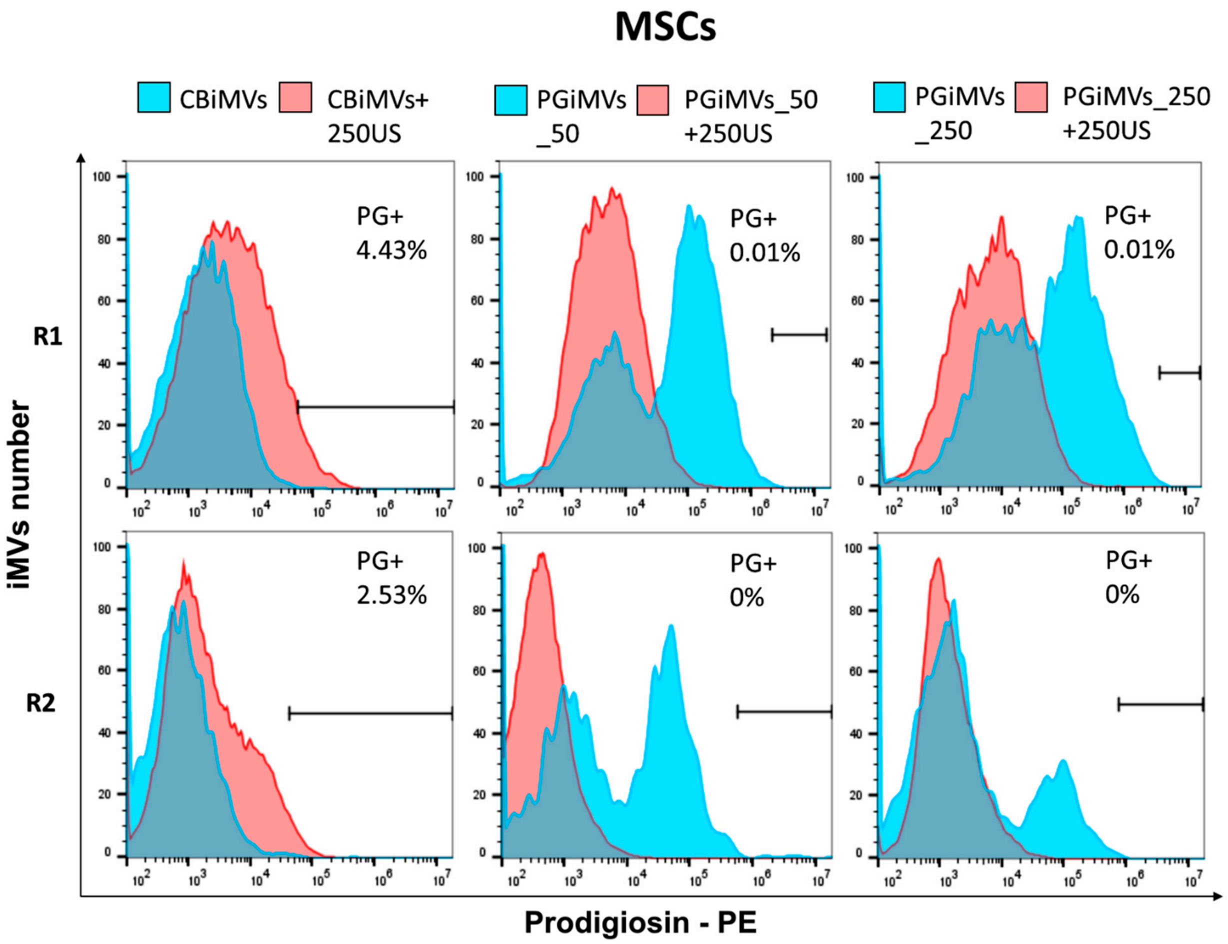

Figure S6. Representative histograms assessed by flow cytometry analysis of prodigiosin content in induced microvesicles obtained from MSCs. The results of two replicates are presented. The percentages reflect the number of induced microvesicles loaded with prodiogisin.
